# A Clinically Practical Nomogram for Predicting Survival in Elderly Patients (≥ 65 Years) With Bladder Urothelial Carcinoma: A Study Based on SEER Database and External Validation

**DOI:** 10.1002/cnr2.70418

**Published:** 2025-12-02

**Authors:** Jufang Wei, Chunmeng Wei, Juan Chen, Xianhui Zhang, Wenpiao Zhao

**Affiliations:** ^1^ Xiangsihu Community Health Service Center Xiangsihu Community Health Service Center Minzu Hospital of Guangxi Zhuang Autonomous Region Nanning China; ^2^ Guangxi Medical University Affiliated Ethnic Hospital Nanning China; ^3^ Department of Urology The First Affiliated Hospital of Guangxi Medical University Nanning China; ^4^ Center for Genomic and Personalized Medicine, Guangxi Key Laboratory for Genomic and Personalized Medicine, Guangxi Collaborative Innovation Center for Genomic and Personalized Medicine, Guangxi Medical University Nanning China; ^5^ Department of Endocrinology Jiangsu Province Hospital of Chinese Medicine Nanjing China; ^6^ Department of Gastroenterology Minzu Hospital of Guangxi Zhuang Autonomous Region Nanning China; ^7^ Department of Urology Maternal and Child Health Hospital of Guangxi Zhuang Autonomous Region Nanning China; ^8^ Guangxi Key Laboratory of Birth Defects Research and Prevention, Guangxi Key Laboratory of Reproductive Health and Birth Defects Prevention Nanning China

**Keywords:** older patients, overall survival, prognosis nomogram, surveillance, epidemiology, and end results, urothelial carcinoma of the bladder

## Abstract

**Background:**

Bladder urothelial carcinoma is a common malignancy in the elderly, yet accurate prognostic tools for older patients remain limited.

**Aims:**

This research aimed to create and validate nomogram predicting overall survival (OS) for elderly patients aged 65 years and older with urothelial carcinoma of the bladder (UCB).

**Methods:**

We sourced 12702 UCB patients diagnosed between 2004 and 2015 from the Surveillance, Epidemiology, and End Results database. The patients were randomized into training (70%) and internal validation (30%) cohorts. In addition, 55 patients from Minzu Hospital of Guangxi Zhuang Autonomous Region between 2012 and 2022 were selected as the external validation cohort. Utilizing univariate and multivariate Cox regression analyses, we devised a nomogram forecasting 1‐, 3‐, and 5‐year OS. Several metrics, including the consistency index (C‐index), calibration plots, area under the receiver operator characteristics (ROC) curve, and decision curve analysis (DCA) were used to validate the validity and clinical utility of the nomogram. Patients were categorized into high‐ and low‐risk profiles, and their survival outcomes were contrasted using the Kaplan‐Meier method and the log‐rank test.

**Results:**

Age, race, marriage, AJCC stage, tumor size, surgery, radiotherapy, and chemotherapy were identified as independent prognostic factors of OS. In the training cohort, internal validation cohort and external validation cohort, the nomogram for predicting OS achieved C‐index values of 0.673 (95% CI: 0.665−0.681), 0.672 (95% CI: 0.660−0.684), and 0.826 (95% CI: 0.732−0.920), respectively. In all cohorts, the calibration plots revealed high consistency between actual and predicted values. The nomogram depicted by ROC and DCA showcased superior stability, predictive value, and clinical applicability for 1, 3−, and 5‐year OS. The risk stratification delineated patients into low‐ and high‐risk brackets, revealing significant prognostic distinctions (*p* ⟨ 0.05).

**Conclusions:**

Based on the SEER database and Chinese data, we developed a reliable nomogram forecasting 1‐, 3‐, and 5‐year OS for older patients with UCB. The nomogram can identified high‐risk and low‐risk patients, aiding clinicians in personalised treatment and prognostic evaluations. This allows high‐risk patients to receive intensive treatment or close follow‐up, while low‐risk patients can avoid overtreatment.

## 1 Introduction

Bladder cancer ranks as the tenth most prevalent cancer globally. Urothelial carcinoma of the bladder (UCB) constitutes about 90% of all bladder cancer cases, with high incidence in Western populations [1, 2]. UCB is divided into muscle‐invasive bladder cancer (MIBC, 25%) and non‐muscle‐invasive bladder cancer (NMIBC, 75%) [3]. Current management strategies—including surgery, radiotherapy, chemotherapy, and targeted therapies [4, 5]—yield limited prognostic improvement. MIBC is highly aggressive; even with radical cystectomy and lymphadenectomy, the 5‐year survival rate is only 38%, and just 6% for those with distant metastases [6]. Despite the high survival rate of NMIBC, the 5‐year postoperative recurrence rate of NMIBC reaches 80%, and 15% progress to MIBC after 5 years [7, 8]. Therefore, the prognosis of patients with MIBC and NMIBC must be considered.

Global bladder cancer incidence continues to rise, escalating healthcare resource demands and economic burdens [9]. One study indicates 991 000 new cases (72.8% more than in 2020) and 397 000 deaths (86.6% more than in 2020) by 2040 [10]. The incidence peaks in the 70–74 age group, directly linking demographic aging to disease burden [11]. The proportion of older people in the population therefore has a significant impact on bladder cancer incidence and mortality.

Traditionally, clinicians use the American Joint Committee on Cancer (AJCC) classification to predict the prognosis of elderly patients with UCB. The AJCC primarily focuses on the tumor's status, regional lymph nodes, and distant metastases. Yet, it overlooks crucial demographic details and treatment approaches, which can be pivotal in determining prognosis. For instance, advanced age post‐radical cystectomy has been linked to less favorable outcomes [12]. This highlights the urgent need for precision medicine tools to improve UCB prognostication.

Nomograms, graphical representations for statistical predictions, can enhance the precision of forecasting outcomes in cancer patients [13]. Their application spans various tumor prognosis studies [14, 15, 16, 17, 18]. Given that nomograms consider a range of vital clinicopathological aspects and offer individualized survival estimates, they've sometimes outperformed the AJCC in accuracy [19]. Thus, our research focuses on devising reasonable nomograms to forecast OS in older patients with UCB, aiding in informed clinical decisions.

However, to the best of our knowledge, while existing nomograms for UCB have been constructed [20, 21, 22], few specifically predict long‐term survival in elderly UCB patients. Therefore, we constructed a predictive model utilizing SEER‐derived clinical data of UCB patients to forecast the 1‐year, 3‐year, and 5‐year overall survival (OS) rates in this population. Concurrently, we collected UCB patient data from a medical institution in China for external validation, with the aim of providing clinical recommendations and insights.

## 2 Materials

**FIGURE 1:**
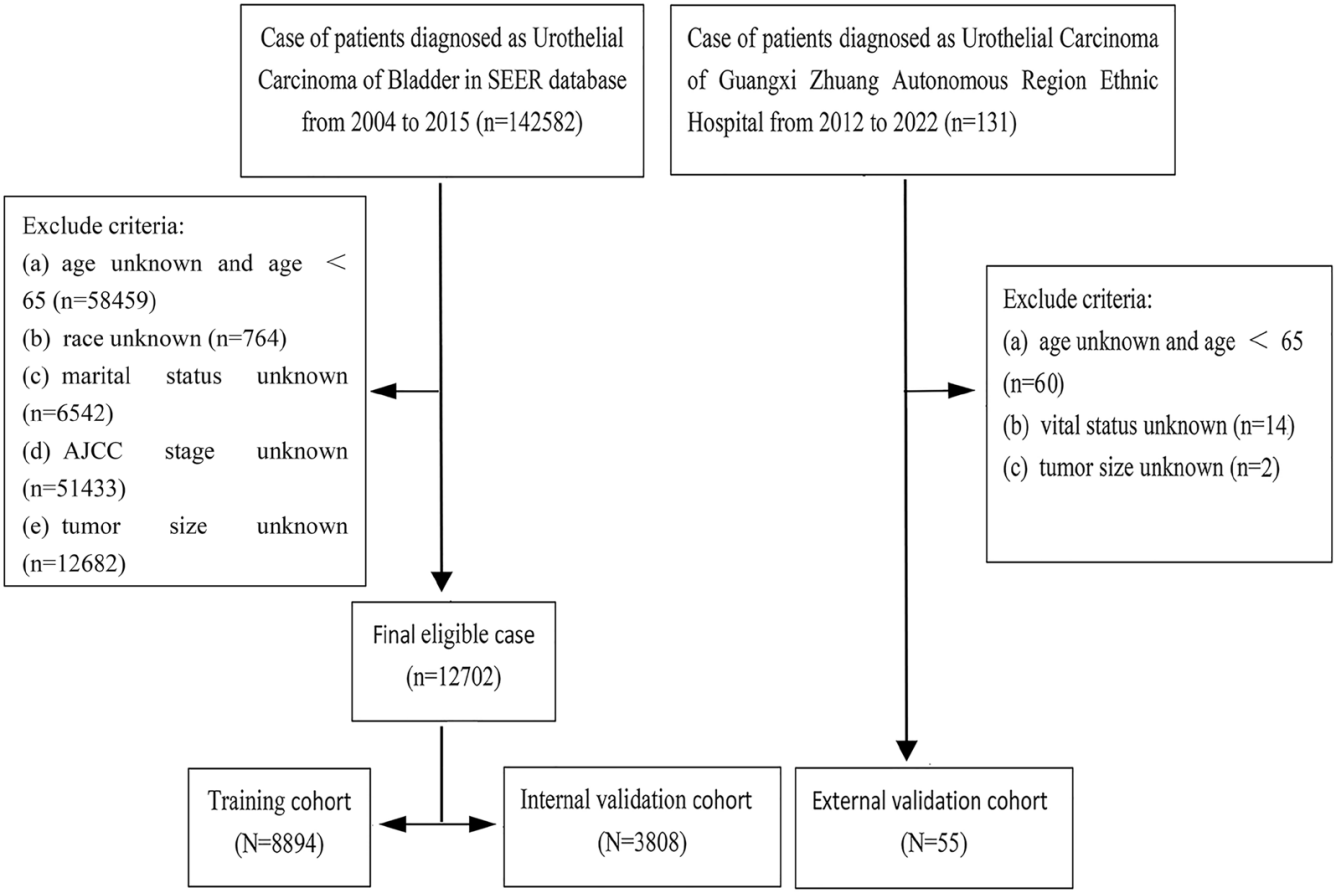
Flowchart for selecting data. SEER.

We sourced patient data from the Surveillance, Epidemiology, and End Results (SEER) database, using SEER*Stat 8.4.0.1 (2000–2017 dataset). In addition, we collected information on patients with a pathological diagnosis of UCB from Guangxi Zhuang Autonomous Region Ethnic Hospital from 2012 to 2022, which was approved by the hospital's ethics approval (No: 2024.41). Inclusion criteria included the tumor‐site ICD‐9: C67.0–C67.9, and the ICD‐O‐3 code was 8130/3. Exclusion criteria included: [1] age < 65; [2] survival time unknown; [3] race unknown; [4] marital status unknown; [5] age unknown; [6] AJCC stage unknown; [7] tumor size unknown. Eligible UCB patients from the SEER database were randomized into the training and internal validation cohorts at a ratio of 7:3. Data from the Guangxi Zhuang Autonomous Region Ethnic Hospital were used as the external validation cohort. The data selection process is detailed in Figure 1. The training cohort facilitated variable filtration and nomogram construction, while the internal validation cohort and external validation cohort served to verify outcomes. We stratified patients by age and risk score using the X‐tile program that accurately determines the precise cutoff point. Therefore, we categorized patients into three age groups: 65–73 years, 74–78 years, and ≥ 79 years. Similarly, UCB patients were categorized into high‐ and low‐risk groups: 0–180.36 and 180.60–384.85 for the training cohort, 0–132.01 and 132.34–336.46 for the internal validation cohort, and 6.58–179.18 and 180.03–190.32 for the external validation cohort.

The variables in the SEER database included demographic characteristics (age, sex, race, and marital status), tumor characteristics (AJCC stage and tumor size), treatment information (surgery, radiotherapy, and chemotherapy), and survival data (survival month and survival status).

We made certain adjustments to some variables: [1] X‐Tile software analysis pinpointed optimal age breakpoints at 73 and 78 years. Thus, we categorized patients into three age groups (65–73 years, 74–78 years, and ≥ 79 years); [2] sex (female and male); [3] race (white, black, and other); [4] marital statuses were grouped into single, married, or separated/divorced/widowed (SDW); [5] AJCC stage (Stage I and II–IV); [6] surgery (no/unknown and yes); [7] radiotherapy (no/unknown and yes); [8] chemotherapy (no/unknown and yes); [9] tumor size (< 30 and ≥ 30 mm). Our analysis primarily focused on OS. OS was defined as the duration from pathological confirmation to any cause of death.

Baseline characteristics of the UCB patient groups were assessed using the Chi‐square test. We initially conducted a univariate Cox regression analysis to pinpoint variables significantly influencing OS (*p* < 0.05). Following this, we included these variables in a multivariate Cox analysis to discern the independent impact of each on survival. Results were presented as hazard ratios (HR) with their associated 95% confidence intervals (95% CI).

The nomogram was developed based on multivariate Cox analysis to predict OS at 1‐, 3‐, and 5‐year. To validate the nomogram, 1000 bootstrap resampling tests were performed on the nomogram. The accuracy of the nomogram was tested via the C‐index. The C‐index value larger than 0.7 indicated a superior prediction model [23]. Calibration plots validated the nomograms; agreement between predicted and observed values, demonstrated by the close fit to the 45° line in the calibration sample, confirmed the model's accuracy. Model performance was evaluated through the ROC curve [24]. The DCA was utilized to evaluate the clinical benefit. Additionally, X‐tile software was utilized to identify an optimal risk score cutoff point, resulting in the stratification of patients into high‐ and low‐risk groups. Survival outcomes for these risk clusters were plotted utilizing the Kaplan–Meier method and contrasted via log‐rank tests.

The data analysis was completed using SPSS version 23.0 (IBM Corp, Armonk, NY), X‐tile software version 3.6.1 (Yale University School of Medicine, New Haven, CT, USA), and R statistical software (version 4.2.1, http://www.r‐project.org). *p*‐value < 0.05 (two‐sided) was deemed statistically significant. We present this article in accordance with the TRIPOD reporting checklist.

## 3 Results

### 3.1 Baseline Characteristics

In this study, we identified 12 702 eligible UCB patients from the SEER database (2004–2015). An external validation cohort comprised 55 patients from Minzu Hospital of Guangxi Zhuang Autonomous Region (2012–2022; 48 males, 7 females). These patients from the SEER database were randomized into a training cohort (*n* = 8894) and an internal validation cohort (*n* = 3808) in a 7:3 ratio. The median OS is 44 months in the SEER database and 48 months in the external validation cohort. In the SEER database, the overall median age was 75 years, with an interquartile range of 70–79 years. A significant proportion were male (78.1%), identified as white (90.2%), and were married (65.4%). Tumors were primarily larger than 30 mm (65.6%). Based on the pathologic tissues, we combined AJCC stage II, AJCC stage III, and AJCC stage IV into AJCC stages II–IV for further analysis. AJCC stage I and AJCC stages II–IV accounted for 66.5% and 33.5%, respectively. Most underwent surgical treatment (98.5%) but declined both radiotherapy (93.6%) and chemotherapy (71.9%). Both cohorts displayed similar demographic and clinical profiles (*p* > 0.05), as detailed in Table 1.

**TABLE 1:** Characteristics of patients with UCB enrolled in the training cohort, internal validation cohort, and external validation cohort.

Characteristics	Training cohort	Internal validation cohort	Total	*p*	External validation cohort
	(*n* = 8894)	(*n* = 3808)	(*n* = 12 702)		(*n* = 55)
Age				0.37	
65–73	3897 (43.8)	1647 (43.3)	5544 (43.6)		25 (45.5)
74–78	2415 (27.2)	1009 (26.5)	3424 (27.0)		14 (25.5)
≥ 79	2582 (29.0)	1152 (30.3)	3734 (29.4)		16 (29.1)
Sex				0.32	
Female	1967 (22.1)	812 (21.3)	2779 (21.9)		7 (12.7)
Male	6927 (77.9)	2996 (78.7)	9923 (78.1)		48 (87.3)
Race				0.50	
White	8043 (90.4)	3418 (89.8)	11 461 (90.2)		—
Black	443 (5.0)	202 (5.3)	645 (5.1)		—
Other	408 (4.6)	188 (4.9)	596 (4.7)		55 (100.0)
Marital status				0.59	
Single	809 (9.1)	350 (9.2)	1159 (9.1)		3 (5.5)
Married	5796 (65.2)	2511 (65.9)	8307 (65.4)		49 (89.1)
SDW	2289 (25.7)	947 (24.9)	3236 (25.5)		3 (5.5)
AJCC stage				0.71	
I	5902 (66.4)	2540 (66.7)	8442 (66.5)		23 (41.8)
II–IV	2992 (33.6)	1268 (33.3)	4260 (33.5)		32 (58.2)
Surgery				0.73	
No/unknown	138 (1.6)	56 (1.5)	194 (1.5)		6 (10.9)
Yes	8756 (98.4)	3752 (98.5)	12 508 (98.5)		49 (89.1)
Radiotherapy				0.38	
No/unknown	8310 (93.4)	3574 (93.9)	11 884 (93.6)		51 (92.7)
Yes	584 (6.6)	234 (6.1)	818 (6.4)		4 (7.3)
Chemotherapy				0.29	
No/unknown	6369 (71.6)	2762 (72.5)	9131 (71.9)		22 (40.0)
Yes	2525 (28.4)	1046 (27.5)	3571 (28.1)		33 (60.0)
Tumor size (mm)				0.53	
< 30	3074 (34.6)	1294 (34.0)	4368 (34.4)		22 (40.0)
≥ 30	5820 (65.4)	2514 (66.0)	8334 (65.6)		33 (60.0)

Abbreviations: AJCC, American Joint Committee on Cancer; SDW, Separated, divorced, and widowed; UCB, Urothelial carcinoma of the bladder.

### 3.2 Construction of Nomogram

**FIGURE 2:**
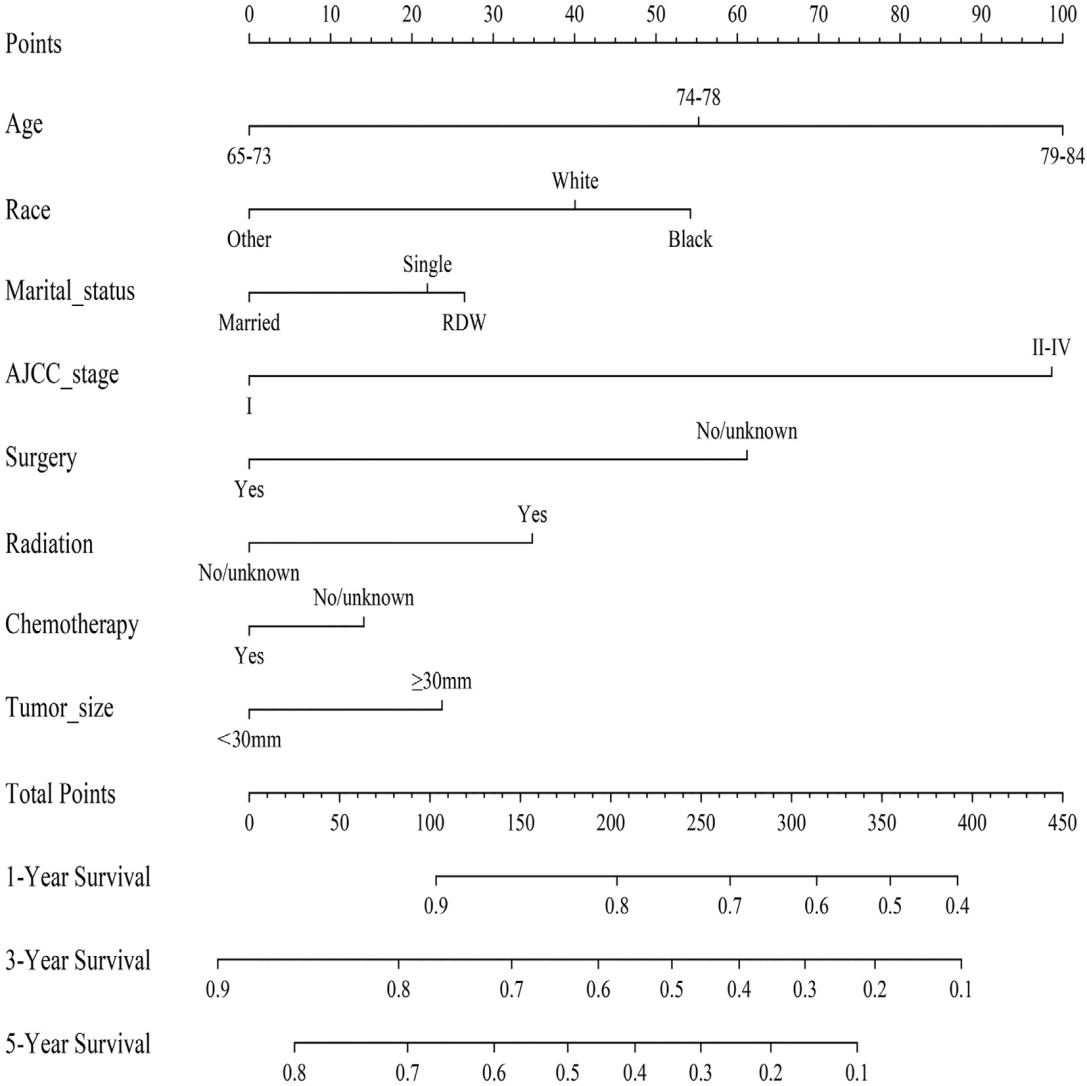
The nomogram to predict the 1‐, 3‐, and 5‐year OS rate of patients with UCB.

Within the training cohort, the univariate analysis and multivariate analysis identified age, race, marital status, AJCC stage, tumor size, surgery, radiotherapy, and chemotherapy as independent prognostic factors influencing OS (Table 2). The independent prognostic factors were utilized to develop a nomogram to assess 1‐, 3‐, and 5‐year probabilities for OS. The most influential variables for OS prognosis were AJCC stage, age, and surgery. In the nomogram, each factor was assigned a specific score. By aggregating these scores, we obtained a comprehensive score that could be used to determine the 1‐, 3‐, and 5‐year likelihood of OS (Figure 2). Additionally, to simplify individual prognosis predictions for UCB patients, we've tabulated the nomogram scores for all variables in Table 3.

**TABLE 2:** Univariate and multivariate Cox analysis of risk factors for OS in UCB patients.

Characteristic	Univariate analyses		Multivariate analyses	
	HR (95% CI)	*p*	HR (95% CI)	*p*
Age				
65–73	Reference		Reference	
74–78	1.537 (1.431–1.650)	< 0.001*	1.513 (1.409–1.625)	< 0.001*
≥ 79	2.192 (2.050–2.343)	< 0.001*	2.117 (1.978–2.266)	< 0.001*
Sex				
Female	Reference			
Male	0.968 (0.905–1.035)	0.34		
Race				
White	Reference		Reference	
Black	1.117 (0.984–1.268)	0.09	1.112 (0.979–1.264)	0.10
Other	0.736 (0.634–0.854)	< 0.001*	0.741 (0.638–0.859)	< 0.001*
Marital status				
Single	Reference		Reference	
Married	0.837 (0.759–0.923)	< 0.001*	0.849 (0.769–0.936)	0.001*
SDW	1.162 (1.047–1.291)	0.005*	1.035 (0.931–1.150)	0.53
AJCC stage				
I	Reference		Reference	
II–IV	2.181 (2.060–2.308)	< 0.001*	2.096 (1.972–2.227)	< 0.001*
Surgery				
No/unknown	Reference		Reference	
Yes	0.593 (0.485–0.724)	< 0.001*	0.632 (0.517–0.773)	< 0.001*
Radiotherapy				
No/unknown	Reference		Reference	
Yes	2.177 (1.976–2.398)	< 0.001*	1.298 (1.167–1.443)	< 0.001*
Chemotherapy				
No/unknown	Reference		Reference	
Yes	1.090 (1.023–1.161)	0.008*	0.900 (0.841–0.963)	0.002*
Tumor size (mm)				
< 30	Reference		Reference	
≥ 30	1.264 (1.191–1.343)	< 0.001*	1.195 (1.124–1.269)	< 0.001*

Abbreviations: AJCC, American Joint Committee on Cancer; CI, Confidence interval; HR, Hazard ratio; OS, Overall survival; SDW, Separated, divorced, and widowed; UCB, Urothelial carcinoma of the bladder.

* *p* < 0.05.

**TABLE 3:** Nomogram‐based scores of all variables of UCB patients.

Variables	Classifications	Scores of OS
Age	65–73	0
	74–78	55
	≥ 79	100
Race	White	40
	Black	54
	Other	0
Marital status	Single	22
	Married	0
	SDW	26
Tumor size	< 30	2
	≥ 30	24
AJCC stage	I	0
	II–IV	99
Surgery	No/unknown	61
	Yes	0
Radiotherapy	No/unknown	0
	Yes	35
Chemotherapy	No/unknown	14
	Yes	0

Abbreviations: AJCC, American Joint Committee on Cancer; OS, Overall survival; SDW, Separated, divorced, and widowed; UCB, Urothelial carcinoma of the bladder.

### 3.3 Evaluation and Validation of the OS Nomogram

**FIGURE 3:**
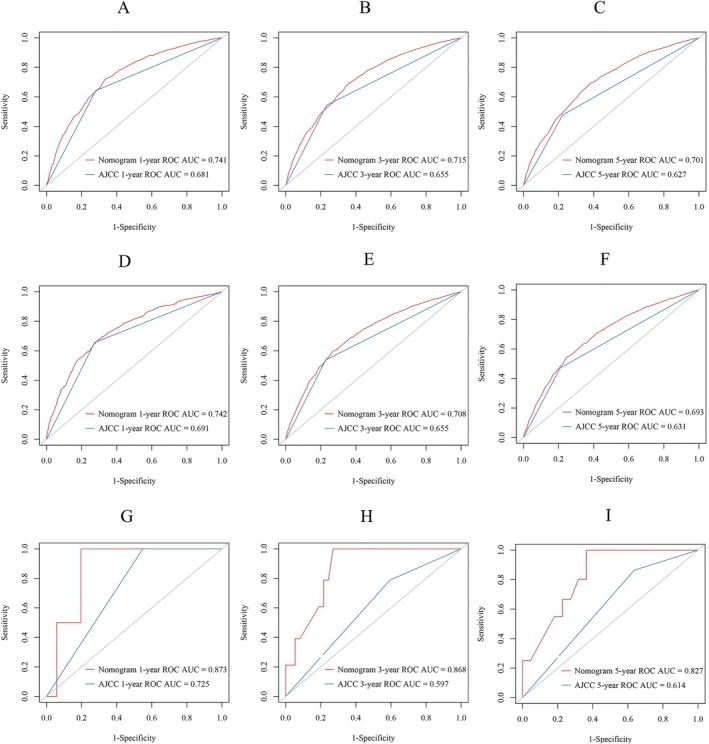
The ROC of OS at 1‐, 3‐, and 5‐year in training cohort (A–C), internal validation cohort (D–F), and external validation cohort (G–I).

**FIGURE 4:**
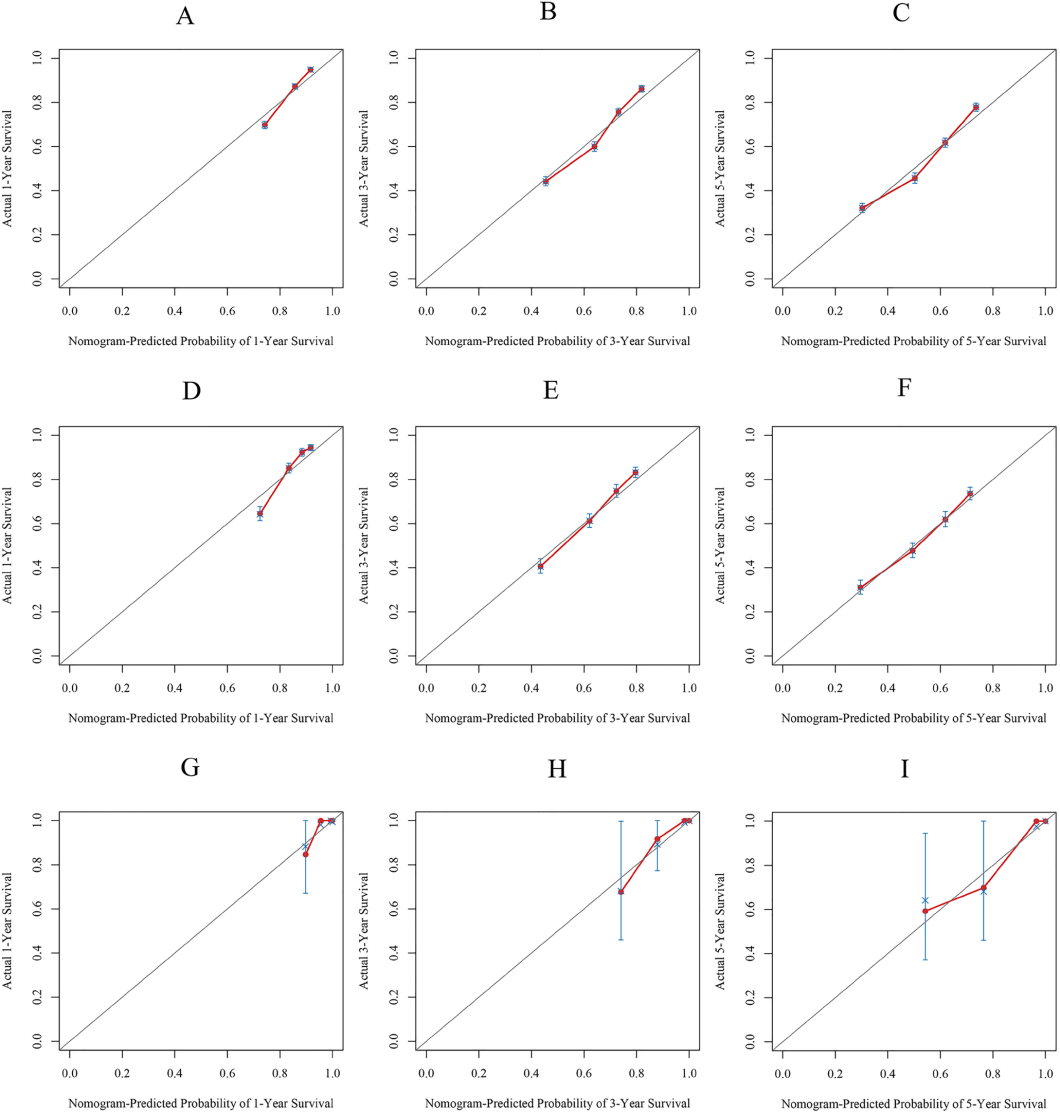
Calibration curve of the nomogram. A, B, and C for 1‐, 3‐, and 5‐year OS in the training cohort; D, E, and F for 1‐, 3‐, and 5‐year OS in the internal validation cohort; G, H, and I for 1‐, 3‐, and 5‐year OS in the external validation cohort.

**FIGURE 5:**
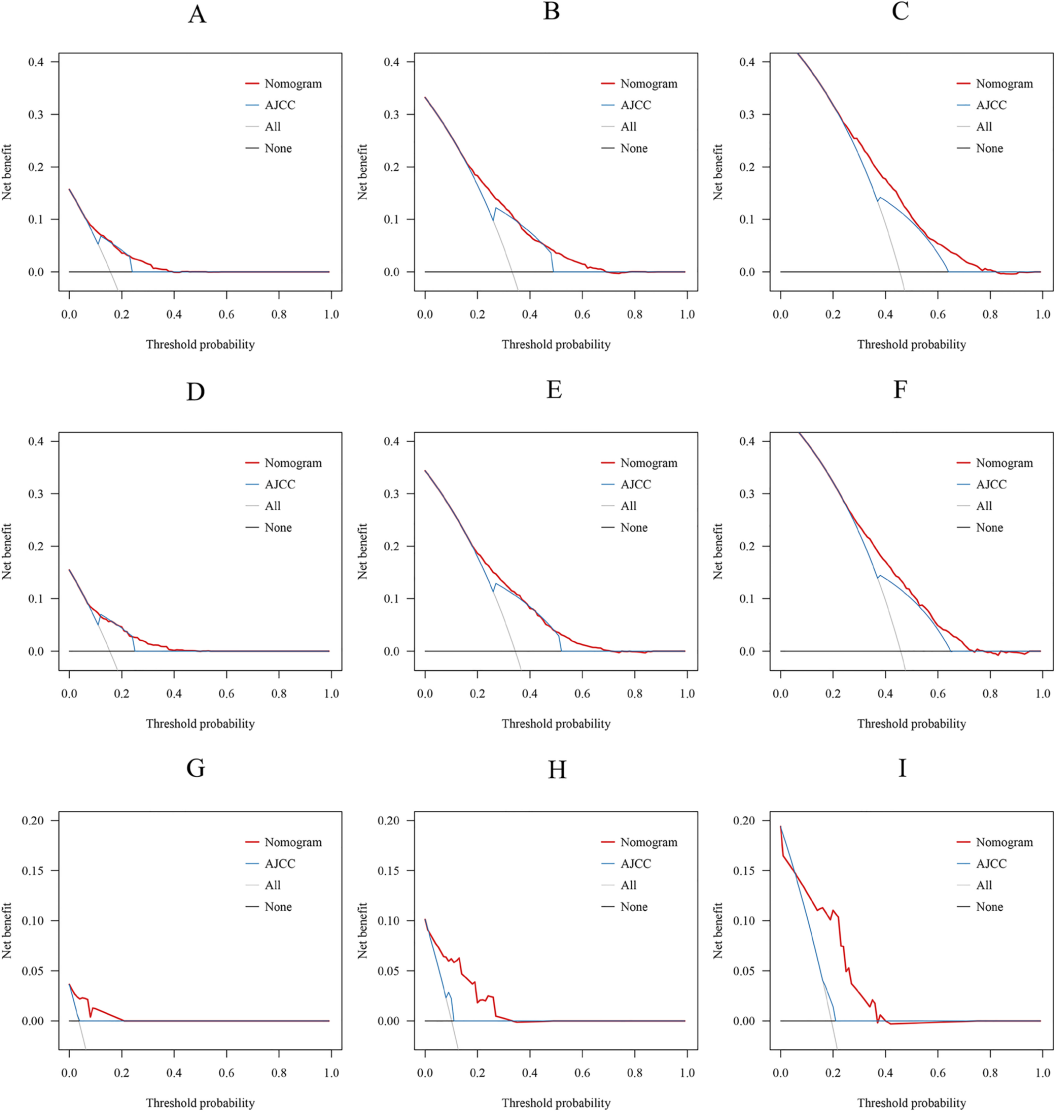
The DCA of OS at 1‐, 3‐, and 5‐year in the training cohort (A–C), internal validation cohort (D–F), and external validation cohort (G–I).

We used C index, calibration curve, ROC values and DCA to assess the performance and clinical utility of the nomogram. In the training cohort, internal validation cohort, and the external validation cohort, the C‐index of OS nomogram was 0.673 (95% CI: 0.665–0.681), 0.672 (95% CI: 0.660–0.684), and 0.826 (95% CI: 0.920–0.732), respectively. Meanwhile, the 1‐, 3‐, and 5‐year ROC values of the training cohort were 0.741, 0.715, and 0.701 for the training cohort, 0.742, 0.708, and 0.693 for the internal validation cohort, and 0.873, 0.868, and 0.827 for the external validation cohort, respectively. The ROC values of the nomogram were all higher than the AJCC staging (Figure 3). The calibration curves for the three cohorts exhibited a strong concordance between the estimates provided by the nomogram and the observed survival probabilities at 1‐, 3‐, and 5‐years, signifying reliable discrimination and calibration capabilities of the models (Figure 4). Furthermore, we assessed the clinical efficacy of our nomogram against the AJCC system. The DCA curves indicated superior predictions for OS using our nomogram, providing enhanced guidance for treatment decisions across a wide range of probability thresholds (Figure 5). Collectively, these findings emphasize that our nomogram can offer precise clinical prognosis compared to those with the AJCC stage.

### 3.4 Risk Rating Based on the Nomogram

**FIGURE 6:**
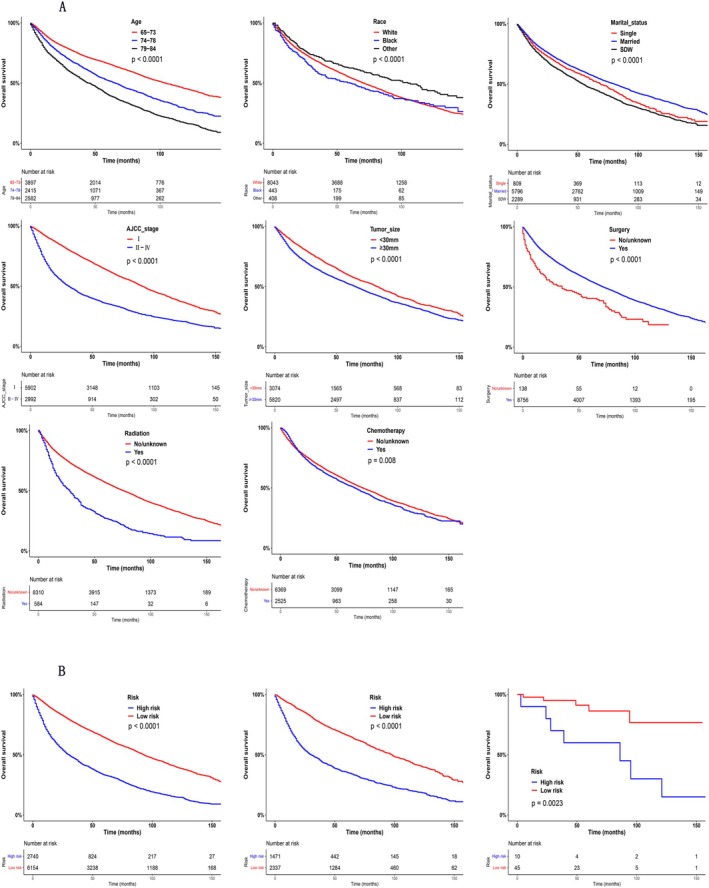
(A) Kaplan–Meier curves for elder patients with UCB according to different independent prognostic factors. The Kaplan–Meier curves for elder patients with UCB according to age, race, marriage, AJCC stage, tumor size, surgery, radiotherapy, chemotherapy. (B) Survival curves of patients in the high‐ and low‐risk groups according to nomogram scores in the training cohort, internal validation cohort, and external validation cohort.

Patient risk categories were defined using X‐tile software to determine optimal score thresholds, stratifying UCB patients into low and high‐risk groups. Specifically, the optimal risk score thresholds were 180.4 for the training cohort, 132.3 for the internal validation cohort, and 179.2 for the external validation cohort. Based on these thresholds, UCB patients were stratified as: 0–180.36 and 180.60–384.85 for the training cohort, 0–132.01 and 132.34–336.46 for the internal validation cohort, and 6.58–179.18 and 180.03–190.32 for the external validation cohort. Using the Kaplan–Meier method, we plotted survival curves for these risk groups as well as for the independent risk factors identified through multivariate analysis, and performed statistical comparisons using the log‐rank test. All survival curves revealed significant disparities between the two groups (*p* < 0.01) (Figure 6).

## 4 Discussion

Currently, in the precision imaging diagnosis of bladder cancer, advancements from VI‐RADS standardization to AI‐assisted analysis have progressively enabled a shift from morphological assessment to functional prediction [25, 26, 27]. This is particularly significant for the clinical management of high‐risk or variant tumors. However, the improvement in bladder cancer prognosis remains limited. According to previous studies, bladder cancer usually occurs in older adults [11], however, few nomograms predict OS in older UCB patients. To address this question, we developed and evaluated a nomogram using a large SEER dataset and an external dataset to estimate the life expectancy of UCB patients. Using a widely accepted approach [28, 29], we randomly divided 12 702 patients into training and internal validation cohorts, and also collected clinical data from 55 Chinese UCB patients as an external validation cohort. Multivariable Cox analysis identified age, race, marital status, AJCC stage, tumor dimensions, surgery, radiotherapy, and chemotherapy as key predictors of OS. Moreover, by stratifying patients into high‐ and low‐risk categories, the model improves prognostic precision for long‐term outcomes, aiding in informed decision‐making. We also found that our model exhibited superior discrimination and accuracy in forecasting the probability of 1‐, 3‐, and 5‐year survival, as evidenced by various performance metrics such as the C‐index, calibration plots, and ROC curves. Additionally, our model demonstrated better clinical applicability than the AJCC stage. In essence, the validated UCB predictive model serves as a valuable tool for understanding patient characteristics and guiding clinical interventions.

We identified age as a critical predictor of UCB prognosis. With aging, a waning immune system can hasten tumor growth and decrease overall survival time. Consistent with this, He et al. reported that reduced functional status and increased comorbidity burden in older patients elevate perioperative mortality and postoperative complications, resulting in a higher risk of bladder cancer‐related mortality [20, 21, 30]. However, conflicting evidence exists: one study found that age ≥ 75 years was not a risk factor for major complications after radical cystectomy despite worse overall survival in older patients [31], while Huang C et al. [32] concluded age may not be an independent predictor. It's worth noting that selection biases, such as patient exclusion, can skew age‐related conclusions. Larger, rigorously designed studies are needed to clarify the role of age in prognosis.

In our study, we found that AJCC stage emerged as the most robust predictor for OS, consistent with the findings reported by Drakaki A. et al. [33]. Enhancing the precision of prognostic models is achievable by combining AJCC stage with other clinical prognostic markers, a sentiment supported in studies across various cancer types [34, 35, 36]. Not surprisingly, the AJCC staging primarily reflects the severity of the tumor.

Existing evidence indicates racial disparities in cancer prognosis, with black patients experiencing worse outcomes than other racial individuals [20, 37, 38]. This may be due to a combination of biology and epidemiology, with some genetic differences between races that may affect patient survival depending on protein synthesis, and may also be related to different racial traditions and cultures [37]. Among elderly UCB patients, married patients demonstrated better OS than single or SDW patients, corroborating prior reports of marital status as a positive prognostic factor [20, 39]. This disparity can be attributed to the better financial capabilities of married patients, facilitating access to superior treatments and care. Furthermore, single bladder cancer patients demonstrate a higher propensity for post‐treatment psychiatric diagnoses than married ones. Notably, a psychiatric diagnosis is often linked to a less favorable bladder cancer prognosis [40], highlighting the interplay between mental health and survival outcomes in UCB patients.

The nomogram incorporates three therapeutic components: surgery, radiotherapy and chemotherapy. Historically, surgery was the primary intervention for UCB patients. In our analysis, those who underwent surgical procedures exhibited notably superior survival rates. A significant proportion of UCB patients show favorable 10‐year metastasis‐free survival after cystectomy [41]. Moreover, individuals undergoing transurethral resection of bladder tumors experience better OS than their counterparts receiving alternate or no surgical treatments [38]. Chemotherapy also plays a pivotal role in enhancing OS for UCB patients. A body of research indicates that patients undergoing chemotherapy typically outlive those who don't [42, 43]. However, our study showed a negative effect of radiotherapy, similar to the results of other studies [20, 44], potentially attributable to treatment‐related toxicities affecting bladder function and adjacent organs, which can compromise quality of life and overall health status, particularly in vulnerable populations [45]. The acute and chronic effects, such as radiation cystitis, proctitis, or bowel dysfunction, may contribute to morbidity that offsets potential oncologic benefits in some cases [46], whereas the Yang study did not show an independent association between radiotherapy and prognosis [47]. It was also recommended that elderly patients be careful when opting for radical radiotherapy, as frail patients may not get much benefit [48]. In light of these findings, we caution against overtreatment with radiotherapy in elderly patients with UCB. Furthermore, our nomogram underscored the detrimental prognosis linked to larger tumor sizes in UCB cases, a finding echoed in various studies [49].

For effective UCB patient management, we classified patients into low‐ and high‐risk categories using the nomogram. The Kaplan–Meier curves and log‐rank tests revealed pronounced disparities between these risk groups. Risk stratification is vital in identifying high‐risk individuals, enabling precise surgical interventions and enhanced monitoring. For example, a 71‐year‐old single white male with stage IV UCB, tumor > 30 mm who underwent surgery and chemotherapy but not radiotherapy. The nomogram assigned a cumulative score of 220, corresponding to predicted 1‐, 3‐, and 5‐year OS rates of 77.62%, 63.43%, and 37.08%, respectively. Given these values, this patient would be categorized as high risk, warranting enhanced care due to his unfavorable prognosis.

The AJCC system has long been the primary tool for prognosticating UCB outcomes. Yet, its limitations are evident. The AJCC often groups UCB patients with varied survival outcomes under a single stage, introducing heterogeneity. This disparity arises as the AJCC system overlooks factors such as age, gender, race, marital status, and treatment type. Our nomogram, which integrated demographic and clinicopathologic features, offered a more comprehensive prognostic tool than the AJCC system, enhancing predictive accuracy and clinical decision support. The DCA underscored the increased clinical utility of our nomogram in predicting survival.

However, our analysis has several limitations that need to be considered. First, our study was a retrospective study and inevitably suffers from a selection bias. Second, most of our cases were sourced from the SEER database, which primarily encompasses data on American patients, thus limiting the generalizability of the nomograms to Asian or European populations. Third, important prognostic factors like underlying disease status, comorbidities, treatment‐related complications, and details regarding the quality of care were not collected [32, 50]. Fourth, the importance of outcomes such as cancer‐specific survival (CSS) and recurrence‐free survival (RFS) was not considered. Atlast, the external validation data for this study came from a single center with a small sample size and validation results may change if the nomogram is validated with a larger external data set. We recommend further studies in the future using randomized, multicenter, large sample size prospective clinical data and collecting more variables for analysis to enhance credibility and optimize nomograms.

## 5 Conclusions

In conclusion, we developed and validated a clinically practical nomogram to predict overall survival (1‐, 3‐, and 5‐year) in elderly UCB patients, integrating demographic and treatment factors beyond conventional staging. The model demonstrated consistent performance in internal and external validation, offering clinicians a personalized prognostic tool. Further prospective multicenter studies are warranted to confirm its generalizability across diverse clinical settings.

## Author Contributions

**Jufang Wei:** writing – original draft, data curation, conceptualization. **Chunmeng Wei:** writing – original draft, data curation. **Juan Chen:** writing – review and editing. **Xianhui Zhang:** writing – original draft, data curation. **Wenpiao Zhao:** writing – review and editing, writing – original draft, visualization, supervision, investigation, data curation.

## Funding

The authors have nothing to report.

## Disclosure

All authors have completed the ICMJE uniform disclosure.

## Ethics Statement

The data for the primary analysis were obtained from the public Surveillance, Epidemiology, and End Results (SEER) database, which does not require ethical approval or individual patient consent. For the external validation cohort from Minzu Hospital of Guangxi Zhuang Autonomous Region, the study was approved by the hospital's Ethics Committee (Approval No. 2024.41).

## Consent

The authors have nothing to report.

## Conflicts of Interest

The authors declare no conflicts of interest.

## Data Availability

The data that support the findings of this study are available on request from the corresponding author. The data are not publicly available due to privacy or ethical restrictions.

## References

[1] A. Richters , K. K. H. Aben , and L. Kiemeney , “The Global Burden of Urinary Bladder Cancer: An Update,” World Journal of Urology 38, no. 8 (2020): 1895–1904. 31676912 10.1007/s00345-019-02984-4 PMC7363726

[2] M. Babjuk , A. Bohle , M. Burger , et al., “EAU Guidelines on Non‐Muscle‐Invasive Urothelial Carcinoma of the Bladder: Update 2016,” European Urology 71, no. 3 (2017): 447–461. 27324428 10.1016/j.eururo.2016.05.041

[3] A. M. Kamat , N. M. Hahn , J. A. Efstathiou , et al., “Bladder Cancer,” Lancet 388, no. 10061 (2016): 2796–2810. 27345655 10.1016/S0140-6736(16)30512-8

[4] N. P. Gupta , R. Goel , A. K. Hemal , et al., “Radical Cystectomy in Septuagenarian Patients With Bladder Cancer,” International Urology and Nephrology 36, no. 3 (2004): 353–358. 15783105 10.1007/s11255-004-0913-2

[5] M. Racioppi , D. D'Agostino , A. Totaro , et al., “Value of Current Chemotherapy and Surgery in Advanced and Metastatic Bladder Cancer,” Urologia Internationalis 88, no. 3 (2012): 249–258. 22354060 10.1159/000335556

[6] K. Wong , F. Abascal , L. Ludwig , et al., “Cross‐Species Oncogenomics Offers Insight Into Human Muscle‐Invasive Bladder Cancer,” Genome Biology 24, no. 1 (2023): 191. 37635261 10.1186/s13059-023-03026-4 PMC10464500

[7] L. Ding , X. Deng , W. Xia , et al., “Development and External Validation of a Novel Nomogram Model for Predicting Postoperative Recurrence‐Free Survival in Non‐Muscle‐Invasive Bladder Cancer,” Frontiers in Immunology 13 (2022): 1070043. 36458001 10.3389/fimmu.2022.1070043 PMC9706099

[8] J. Y. Teoh , A. M. Kamat , P. C. Black , P. Grivas , S. F. Shariat , and M. Babjuk , “Recurrence Mechanisms of Non‐Muscle‐Invasive Bladder Cancer—A Clinical Perspective,” Nature Reviews. Urology 19, no. 5 (2022): 280–294. 35361927 10.1038/s41585-022-00578-1

[9] L. M. C. van Hoogstraten , A. Vrieling , A. G. van der Heijden , M. Kogevinas , A. Richters , and L. A. Kiemeney , “Global Trends in the Epidemiology of Bladder Cancer: Challenges for Public Health and Clinical Practice,” Nature Reviews. Clinical Oncology 20, no. 5 (2023): 287–304. 10.1038/s41571-023-00744-3 36914746

[10] Y. Zhang , H. Rumgay , M. Li , H. Yu , H. Pan , and J. Ni , “The Global Landscape of Bladder Cancer Incidence and Mortality in 2020 and Projections to 2040,” Journal of Global Health 13 (2023): 04109. 37712386 10.7189/jogh.13.04109 PMC10502766

[11] H. Zi , S. H. He , X. Y. Leng , et al., “Global, Regional, and National Burden of Kidney, Bladder, and Prostate Cancers and Their Attributable Risk Factors, 1990‐2019,” Military Medical Research 8, no. 1 (2021): 60. 34819142 10.1186/s40779-021-00354-z PMC8611255

[12] M. E. Nielsen , S. F. Shariat , P. I. Karakiewicz , et al., “Advanced Age Is Associated With Poorer Bladder Cancer‐Specific Survival in Patients Treated With Radical Cystectomy,” European Urology 51, no. 3 (2007): 699–706. 17113703 10.1016/j.eururo.2006.11.004

[13] V. P. Balachandran , M. Gonen , J. J. Smith , and R. P. DeMatteo , “Nomograms in Oncology: More Than Meets the Eye,” Lancet Oncology 16, no. 4 (2015): e173–e180. 25846097 10.1016/S1470-2045(14)71116-7 PMC4465353

[14] J. Wu , H. Zhang , L. Li , et al., “A Nomogram for Predicting Overall Survival in Patients With Low‐Grade Endometrial Stromal Sarcoma: A Population‐Based Analysis,” Cancer Communications 40, no. 7 (2020): 301–312. 32558385 10.1002/cac2.12067 PMC7365459

[15] L. Han , W. Dai , S. Mo , et al., “Nomogram of Conditional Survival Probability of Long‐Term Survival for Metastatic Colorectal Cancer: A Real‐World Data Retrospective Cohort Study From SEER Database,” International Journal of Surgery 92 (2021): 106013. 34233209 10.1016/j.ijsu.2021.106013

[16] H. Liu , Z. Li , Q. Zhang , et al., “Multi‐Institutional Development and Validation of a Nomogram to Predict Prognosis of Early‐Onset Gastric Cancer Patients,” Frontiers in Immunology 13 (2022): 1007176. 36148218 10.3389/fimmu.2022.1007176 PMC9488636

[17] J. Wang , J. Tang , T. Chen , et al., “A Web‐Based Prediction Model for Overall Survival of Elderly Patients With Early Renal Cell Carcinoma: A Population‐Based Study,” Journal of Translational Medicine 20, no. 1 (2022): 90. 35164796 10.1186/s12967-022-03287-w PMC8845298

[18] X. Zhan , M. Jiang , W. Deng , X. Liu , L. Chen , and B. Fu , “Development and Validation of a Prognostic Nomogram for Predicting Cancer‐Specific Survival in Patients With Lymph Node Positive Bladder Cancer: A Study Based on SEER Database,” Frontiers in Oncology 12 (2022): 789028. 35186736 10.3389/fonc.2022.789028 PMC8851926

[19] X. Zhan , L. Chen , M. Jiang , and B. Fu , “Development and Validation of a Prognostic Nomogram for Predicting Overall Survival for T1 High‐Grade Patients After Radical Cystectomy: A Study Based on SEER,” International Journal of General Medicine 15 (2022): 3753–3765. 35411173 10.2147/IJGM.S354740 PMC8994665

[20] W. Wang , J. Liu , and L. Liu , “Development and Validation of a Prognostic Model for Predicting Overall Survival in Patients With Bladder Cancer: A SEER‐Based Study,” Frontiers in Oncology 11 (2021): 692728. 34222021 10.3389/fonc.2021.692728 PMC8247910

[21] H. He , T. Liu , D. Han , et al., “Incidence Trends and Survival Prediction of Urothelial Cancer of the Bladder: A Population‐Based Study,” World Journal of Surgical Oncology 19, no. 1 (2021): 221. 34311753 10.1186/s12957-021-02327-x PMC8314553

[22] J. Ji , Y. Yao , L. Sun , Q. Yang , and G. Zhang , “Development and Validation of a Preoperative Nomogram to Predict Lymph Node Metastasis in Patients With Bladder Urothelial Carcinoma,” Journal of Cancer Research and Clinical Oncology 149, no. 12 (2023): 10911–10923. 37318590 10.1007/s00432-023-04978-7 PMC10423104

[23] H. Higuchi , “Lattice Swelling With the Selective Digestion of Elastic Components in Single‐Skinned Fibers of Frog Muscle,” Biophysical Journal 52, no. 1 (1987): 29–32. 3496923 10.1016/S0006-3495(87)83185-5 PMC1329980

[24] A. N. Kamarudin , T. Cox , and R. Kolamunnage‐Dona , “Time‐Dependent ROC Curve Analysis in Medical Research: Current Methods and Applications,” BMC Medical Research Methodology 17, no. 1 (2017): 53. 28388943 10.1186/s12874-017-0332-6 PMC5384160

[25] V. Panebianco , Y. Narumi , E. Altun , et al., “Multiparametric Magnetic Resonance Imaging for Bladder Cancer: Development of VI‐RADS (Vesical Imaging‐Reporting and Data System),” European Urology 74, no. 3 (2018): 294–306. 29755006 10.1016/j.eururo.2018.04.029 PMC6690492

[26] Y. Arita , S. Yoshida , K. Shigeta , et al., “Diagnostic Value of the Vesical Imaging‐Reporting and Data System in Bladder Urothelial Carcinoma With Variant Histology,” European Urology Oncology 6, no. 1 (2023): 99–102. 35933266 10.1016/j.euo.2022.07.006

[27] Y. Arita , T. C. Kwee , O. Akin , et al., “Multiparametric MRI and Artificial Intelligence in Predicting and Monitoring Treatment Response in Bladder Cancer,” Insights Into Imaging 16, no. 1 (2025): 7. 39747744 10.1186/s13244-024-01884-5 PMC11695553

[28] M. Fujita , K. Nagashima , S. Takahashi , K. Suzuki , T. Fujisawa , and A. Hata , “Handheld Flow Meter Improves COPD Detectability Regardless of Using a Conventional Questionnaire: A Split‐Sample Validation Study,” Respirology 25, no. 2 (2020): 191–197. 31188538 10.1111/resp.13602

[29] A. Gallina , F. K. Chun , A. Briganti , et al., “Development and Split‐Sample Validation of a Nomogram Predicting the Probability of Seminal Vesicle Invasion at Radical Prostatectomy,” European Urology 52, no. 1 (2007): 98–105. 17267098 10.1016/j.eururo.2007.01.060

[30] K. Chamie , M. S. Litwin , J. C. Bassett , et al., “Recurrence of High‐Risk Bladder Cancer: A Population‐Based Analysis,” Cancer 119, no. 17 (2013): 3219–3227. 23737352 10.1002/cncr.28147 PMC3773281

[31] H. Wang , H. Huang , M. Shang , H. Hao , and Z. Xi , “Comparative Study of Perioperative and Oncological Outcomes Between Elderly Patients and Younger Patients Who Received Radical Cystectomy and Pelvic Lymph Node Dissection: A Single‐Center Retrospective Study,” Cancer Management and Research 14 (2022): 603–613. 35210857 10.2147/CMAR.S350587 PMC8857955

[32] C. Huang , W. Zhou , P. Song , and N. Yuan , “Comparison of Different Prognostic Models for Predicting Cancer‐Specific Survival in Bladder Transitional Cell Carcinoma,” Future Oncology 15, no. 8 (2019): 851–864. 30657341 10.2217/fon-2018-0695

[33] A. Drakaki , A. Pantuck , S. K. Mhatre , et al., “Real‐World Outcomes and Prognostic Indicators Among Patients With High‐Risk Muscle‐Invasive Urothelial Carcinoma,” Urologic Oncology 39, no. 1 (2021): e15–e76. 10.1016/j.urolonc.2020.07.011 32778476

[34] M. L. Lecklitner , “Hepatobiliary Scintigraphy: Causes of Prolonged Retention in Hepatic Parenchyma,” Seminars in Nuclear Medicine 14, no. 3 (1984): 262–263. 6474197 10.1016/s0001-2998(84)80019-7

[35] H. Fukuda , M. Nagai , M. Kobayashi , H. Miyairi , and A. Muramatsu , “The Rotational Performance of Dental High‐Speed Handpieces,” Tokyo Ika Shika Daigaku Iyo Kizai Kenkyusho Hokoku 15 (1981): 61–68. 7051207

[36] T. Hu , Z. Chen , M. Hou , and K. Lin , “Overall and Cancer‐Specific Survival in Patients With Breast Paget Disease: A Population‐Based Study,” Experimental Biology and Medicine (Maywood, N.J.) 247, no. 3 (2022): 187–199. 10.1177/15353702211056264 PMC8851527 34842487

[37] W. Fang , Z. Y. Yang , T. Y. Chen , X. F. Shen , and C. Zhang , “Ethnicity and Survival in Bladder Cancer: A Population‐Based Study Based on the SEER Database,” Journal of Translational Medicine 18, no. 1 (2020): 145. 32228610 10.1186/s12967-020-02308-w PMC7106682

[38] J. Wang , Y. Wu , W. He , B. Yang , and X. Gou , “Nomogram for Predicting Overall Survival of Patients With Bladder Cancer: A Population‐Based Study,” International Journal of Biological Markers 35, no. 2 (2020): 29–39. 10.1177/1724600820907605 32312147

[39] L. Tao , X. Pan , L. Zhang , et al., “Marital Status and Prognostic Nomogram for Bladder Cancer With Distant Metastasis: A SEER‐Based Study,” Frontiers in Oncology 10 (2020): 586458. 33194738 10.3389/fonc.2020.586458 PMC7654226

[40] U. Jazzar , S. Yong , Z. Klaassen , et al., “Impact of Psychiatric Illness on Decreased Survival in Elderly Patients With Bladder Cancer in the United States,” Cancer 124, no. 15 (2018): 3127–3135. 29660813 10.1002/cncr.31404 PMC6097900

[41] L. Cheng , A. L. Weaver , B. C. Leibovich , et al., “Predicting the Survival of Bladder Carcinoma Patients Treated With Radical Cystectomy,” Cancer 88, no. 10 (2000): 2326–2332. 10820355 10.1002/(sici)1097-0142(20000515)88:10<2326::aid-cncr17>3.0.co;2-t

[42] M. Froehner , R. Koch , U. Heberling , et al., “Decreased Overall and Bladder Cancer‐Specific Mortality With Adjuvant Chemotherapy After Radical Cystectomy: Multivariable Competing Risk Analysis,” European Urology 69, no. 6 (2016): 984–987. 26194042 10.1016/j.eururo.2015.06.053

[43] G. Gandaglia , I. Popa , F. Abdollah , et al., “The Effect of Neoadjuvant Chemotherapy on Perioperative Outcomes in Patients Who Have Bladder Cancer Treated With Radical Cystectomy: A Population‐Based Study,” European Urology 66, no. 3 (2014): 561–568. 24486024 10.1016/j.eururo.2014.01.014

[44] W. Mao , B. Ma , X. Huang , et al., “Which Treatment Is Best for Patients With AJCC Stage IV Bladder Cancer?,” International Urology and Nephrology 51, no. 7 (2019): 1145–1156. 30949839 10.1007/s11255-019-02105-5

[45] T. Iwata , S. Kimura , M. Abufaraj , et al., “The Role of Adjuvant Radiotherapy After Surgery for Upper and Lower Urinary Tract Urothelial Carcinoma: A Systematic Review,” Urologic Oncology 37, no. 10 (2019): 659–671. 31255542 10.1016/j.urolonc.2019.05.021

[46] A. N. Viswanathan , L. J. Lee , J. R. Eswara , et al., “Complications of Pelvic Radiation in Patients Treated for Gynecologic Malignancies,” Cancer 120, no. 24 (2014): 3870–3883. 25056522 10.1002/cncr.28849

[47] Z. Yang , Y. Bai , M. Liu , X. Hu , and P. Han , “Development and Validation of a Prognostic Nomogram for Predicting Cancer‐Specific Survival After Radical Cystectomy in Patients With Bladder Cancer: A Population‐Based Study,” Cancer Medicine 9, no. 24 (2020): 9303–9314. 33063464 10.1002/cam4.3535 PMC7774742

[48] C. Wujanto , J. Tey , D. Chia , et al., “Radical Radiotherapy in Older Patients With Muscle Invasive Bladder Cancer,” Journal of Geriatric Oncology 10, no. 2 (2019): 292–297. 30630748 10.1016/j.jgo.2018.10.015

[49] M. Abudurexiti , J. Ma , Y. Li , et al., “Clinical Outcomes and Prognosis Analysis of Younger Bladder Cancer Patients,” Current Oncology 29, no. 2 (2022): 578–588. 35200552 10.3390/curroncol29020052 PMC8870851

[50] M. Morioka , Y. Jo , Y. Furukawa , et al., “Prognostic Factors for Survival and Bladder Recurrence in Transitional Cell Carcinoma of the Upper Urinary Tract,” International Journal of Urology 8, no. 7 (2001): 366–373. 11442658 10.1046/j.1442-2042.2001.00315.x

